# Correction: A modified TurboID approach identifies tissue-specific centriolar components in *C*. *elegans*

**DOI:** 10.1371/journal.pgen.1010645

**Published:** 2023-02-13

**Authors:** Elisabeth Holzer, Cornelia Rumpf-Kienzl, Sebastian Falk, Alexander Dammermann

Panel E of Fig 2 was assembled with incorrect insets. The authors apologize for the error and have provided a corrected version of Fig 2 here.

**Fig 2:**
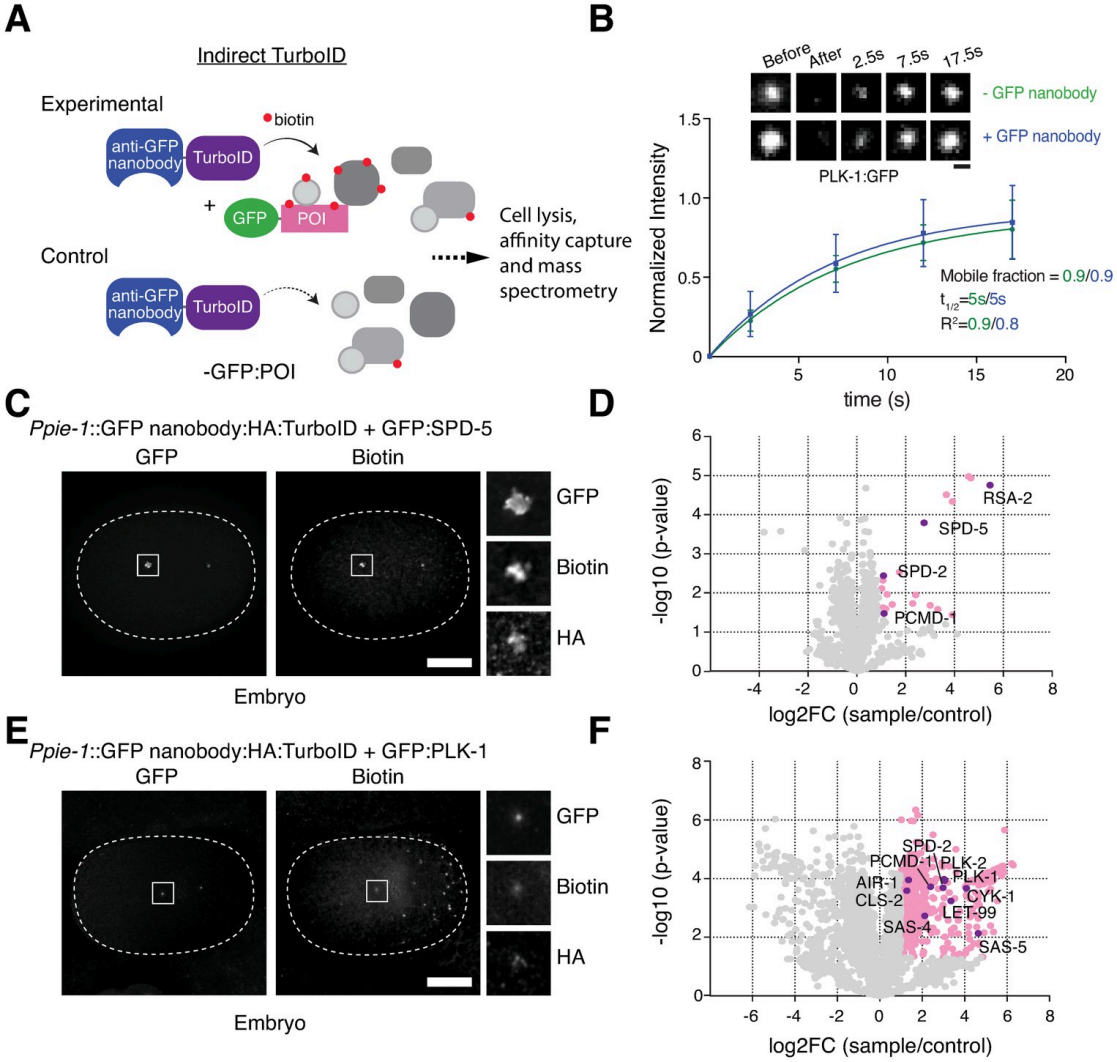
GFP nanobody-directed TurboID as an improved method for proximity-dependent labeling. (**A**) Schematic of indirect TurboID method, whereby the biotin ligase is targeted to an endogenous GFP fusion via a GFP nanobody [41]. Note that experimental and control strains utilize the same TurboID fusion, which may be expressed under a tissue or developmental stage-specific or inducible promoter, while the target protein is potentially expressed in a wide array of tissues and cell types. (**B**) GFP nanobody addition does not perturb PLK-1 mobility. Selected images and quantitation for fluorescence recovery after photobleaching (FRAP) analysis performed on PLK-1:GFP at centrosomes in prometaphase-stage embryos in the presence (n = 16 animals) or absence (n = 13) of the GFP nanobody:TurboID fusion. (**C**) Indirect TurboID applied to SPD-5. Immunofluorescence micrograph of early embryo from strain co-expressing a GFP nanobody:HA:TurboID fusion under the germline promoter *pie-1* and endogenously GFP-tagged SPD-5 stained for GFP, biotin (streptavidin) and HA. Biotinylation signal is observed at centrosomes coincident with GFP:SPD-5 and the TurboID fusion. (**D**) Result of LC-MS/MS analysis for indirect TurboID on SPD-5 from mixed-stage embryos. Volcano plot of -log10 p-values against log2 fold change (sample/control). Significantly enriched proteins (Log2 enrichment >1, p-value <0.05) are indicated in pink, with selected proteins highlighted. Compare Fig 1D. See also S1 Table and S2E Fig. (**E**) Indirect TurboID applied to PLK-1. Immunofluorescence micrograph of early embryo from strain co-expressing a GFP nanobody:HA:TurboID fusion under the germline promoter *pie-1* and endogenously GFP-tagged PLK-1 stained for GFP, biotin (streptavidin) and HA. Biotinylation signal is observed at centrosomes coincident with PLK-1:GFP and the TurboID fusion. (**F**) Result of LC-MS/MS analysis for indirect TurboID on PLK-1 from mixed-stage embryos. Compare Fig 1F. See also S1 Table and S2E Fig. Scale bars are 1μm (B), 10μm (C, E).

## References

[1] Holzer E, Rumpf-Kienzl C, Falk S, Dammermann A (2022) A modified TurboID approach identifies tissue-specific centriolar components in *C*. *elegans*. PLoS Genet 18(4): e1010150. 10.1371/journal.pgen.1010150 35442950 PMC9020716

