# Supplementary figures and images for: A modified TurboID approach identifies tissue-specific centriolar components in C. elegans
Source: PLoS Genet. 2022 Apr 20;18(4):e1010150. doi: 10.1371/journal.pgen.1010150 (PMC9020716; doi:10.1371/journal.pgen.1010150)

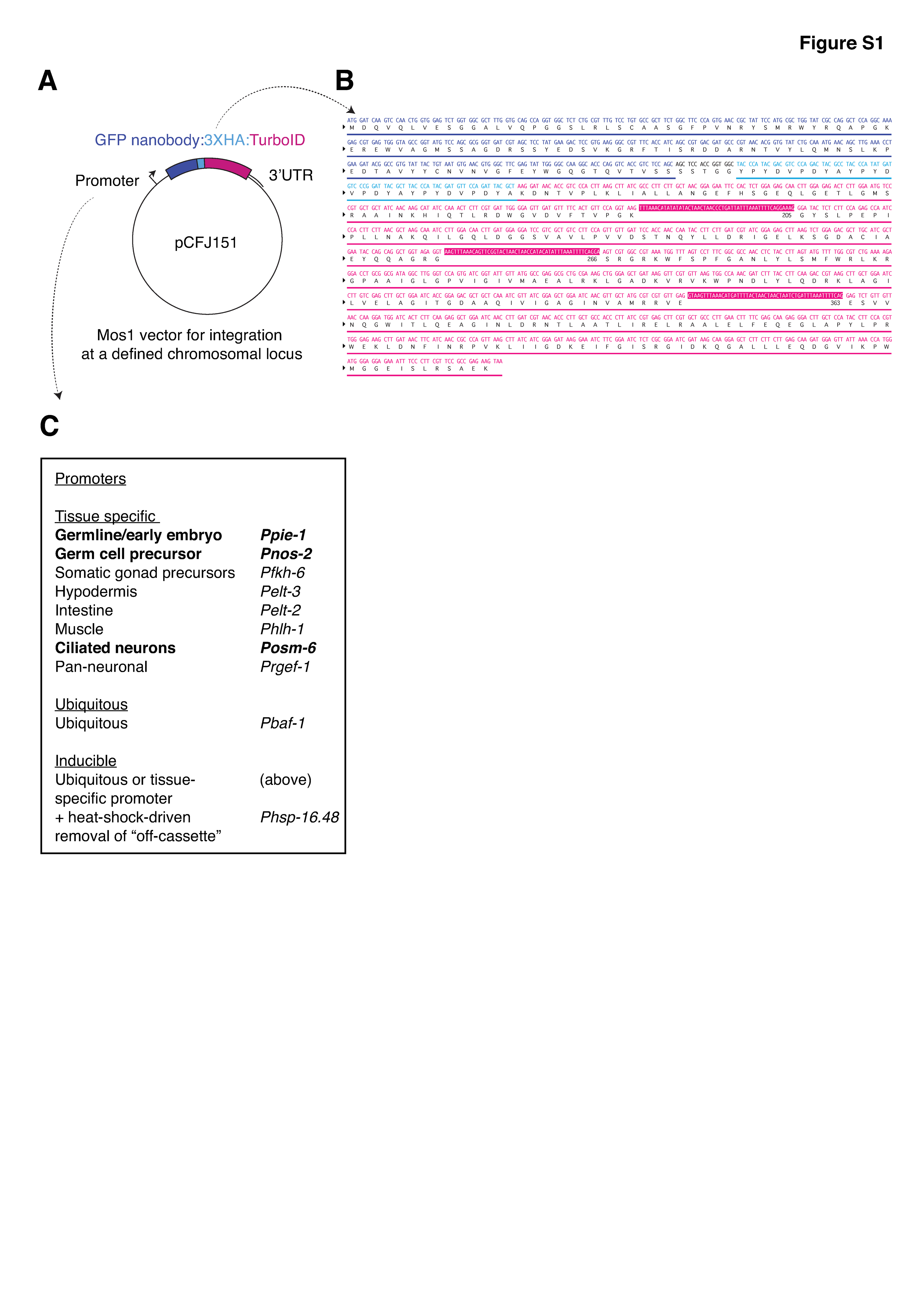

Supplement: S1 Fig — (A) Schematic of the indirect TurboID construct developed in this study. A GFP nanobody:HA:TurboID cassette is expressed under a ubiquitous or tissue/cell-specific promoter and 3’ regulatory sequences. The vector backbone is based on pCFJ151 for Mos1 transposon-mediated integration at a defined chromosomal locus [26], sites for which have been established on all five autosomes in C. elegans [95]. (B) DNA sequence of the GFP nanobody:HA:TurboID cassette and conceptual translation. (C) Promoters for ubiquitous, tissue/cell-specific and inducible expression in C. elegans, selected for the low expression required for TurboID. Promoters in bold have been used and validated in this study. For inducible expression, a strategy such as using FLP-recombinase under the control of the Phsp-16.48 heat shock promoter to excise a repressive “off-element” [99] avoids the variability and overexpression associated with the use of the heat-shock promoter alone and additionally confers the potential for tissue-specific inducible expression not explored in this study. (TIF) [file pgen.1010150.s001.tif]

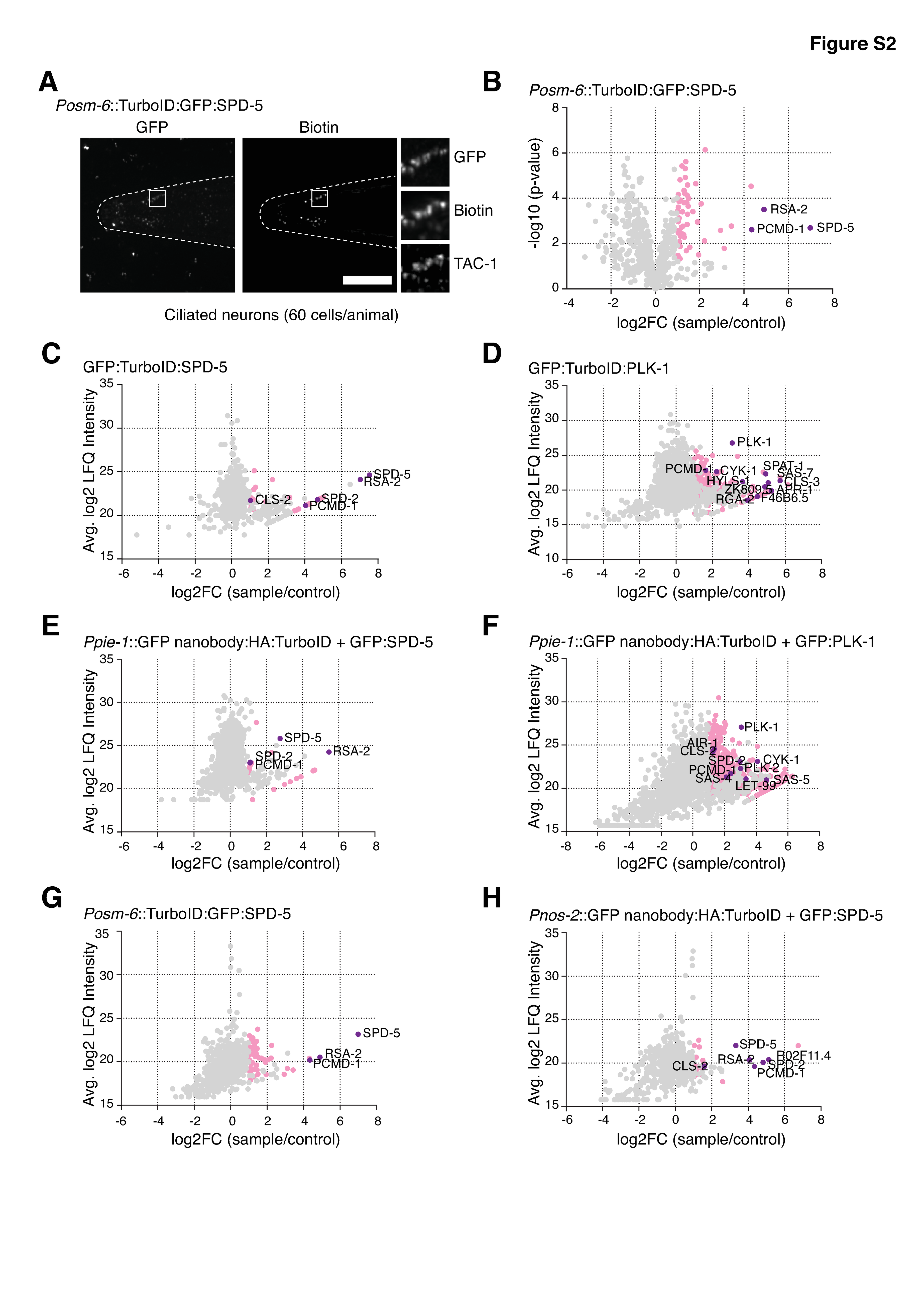

Supplement: S2 Fig — (A) Direct TurboID applied to SPD-5 in ciliated neurons. Immunofluorescence micrograph of head of L1 larva from strain expressing TurboID:GFP:SPD-5 under the ciliated neuron-specific promoter osm-6 stained for GFP, biotin (streptavidin) and TAC-1 as a PCM countermarker. Biotinylation signal is observed at the ciliary base coincident with GFP:SPD-5/TAC-1 signal. (B) Result of LC-MS/MS analysis for direct TurboID on SPD-5 in ciliated neurons. Volcano plot of -log10 p-values against log2 fold change (sample/control). Significantly enriched proteins (Log2 enrichment >1, p-value <0.05) are indicated in pink, with selected proteins highlighted. Comparison with indirect TurboID presented in Fig 3C. See also S1 Table. (C-H) Volcano plots of average log2 LFQ intensity (sample) against log2 fold change (sample/control) for direct (C, D) and indirect (E, F) TurboID on SPD-5 and PLK-1 in embryos, direct TurboID on SPD-5 in ciliated neurons (G) and indirect TurboID on SPD-5 in germ cell precursors (H). Significantly enriched proteins (Log2 enrichment >1, p-value <0.05) are indicated in pink, with selected proximity interactors highlighted. Note that interactors are present at much lower levels in the sample compared to endogenously biotinylated proteins, highlighting the importance of proper sample normalization. Scale bar in A is 10μm. (TIF) [file pgen.1010150.s002.tif]

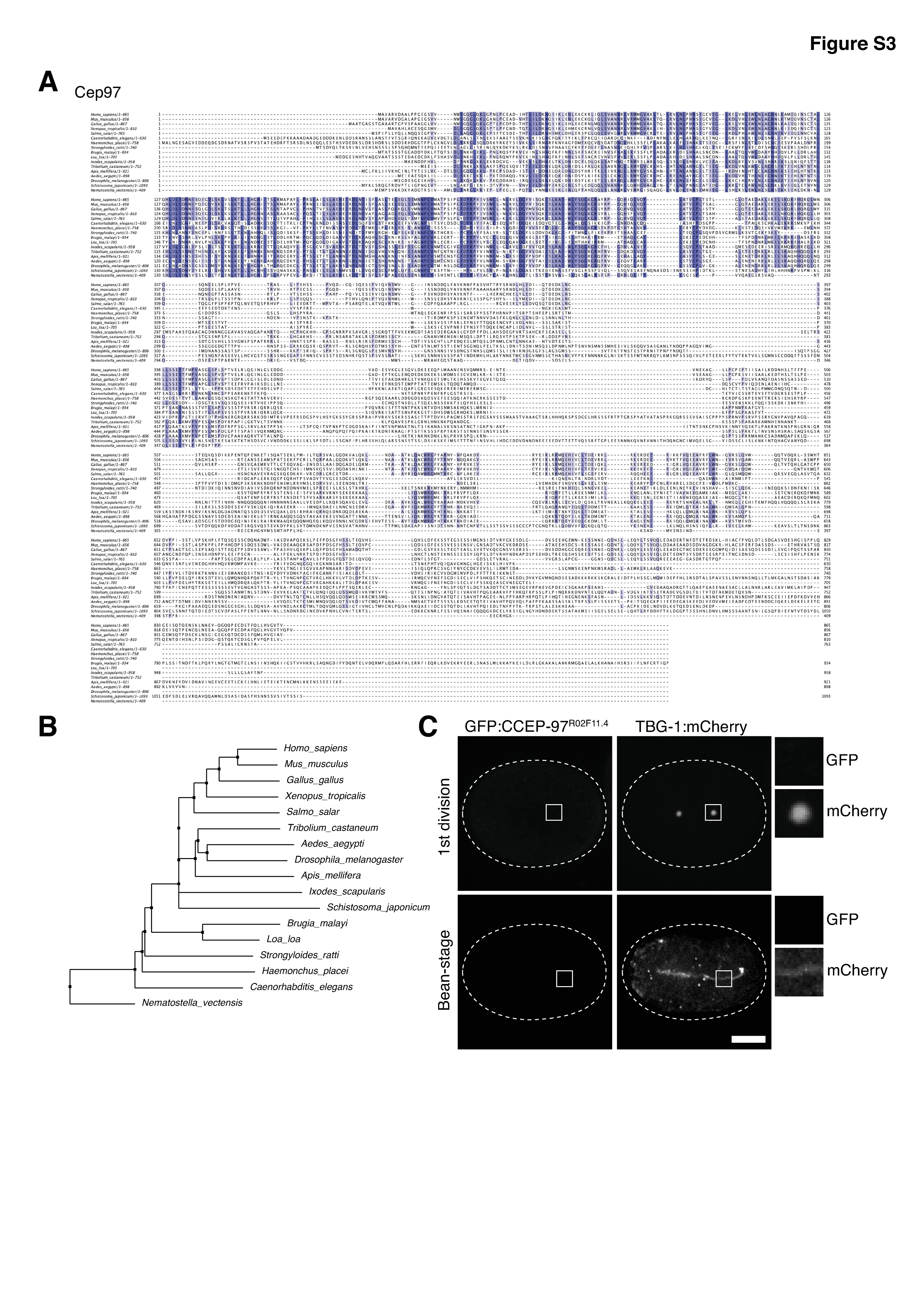

Supplement: S3 Fig — (A, B) Multiple sequence alignment (A) and neighbor joining phylogenetic tree (B) of selected Cep97 orthologs. Accession numbers are provided in S2 Table. Note that tree largely reflects pattern of evolutionary divergence. (C) Endogenous promoter GFP fusion of CCEP-97 does not localize to the mitotic centrosome in the early embryo or the non-centrosomal microtubule-organizing center in the intestinal primordium of bean-stage embryos (~360min after fertilization), both marked by TBG-1:mCherry. GFP images scaled to centrosomal signal at germ cell precursors in L1 stage larvae. Scale bar in C is 10μm. (TIF) [file pgen.1010150.s003.tif]

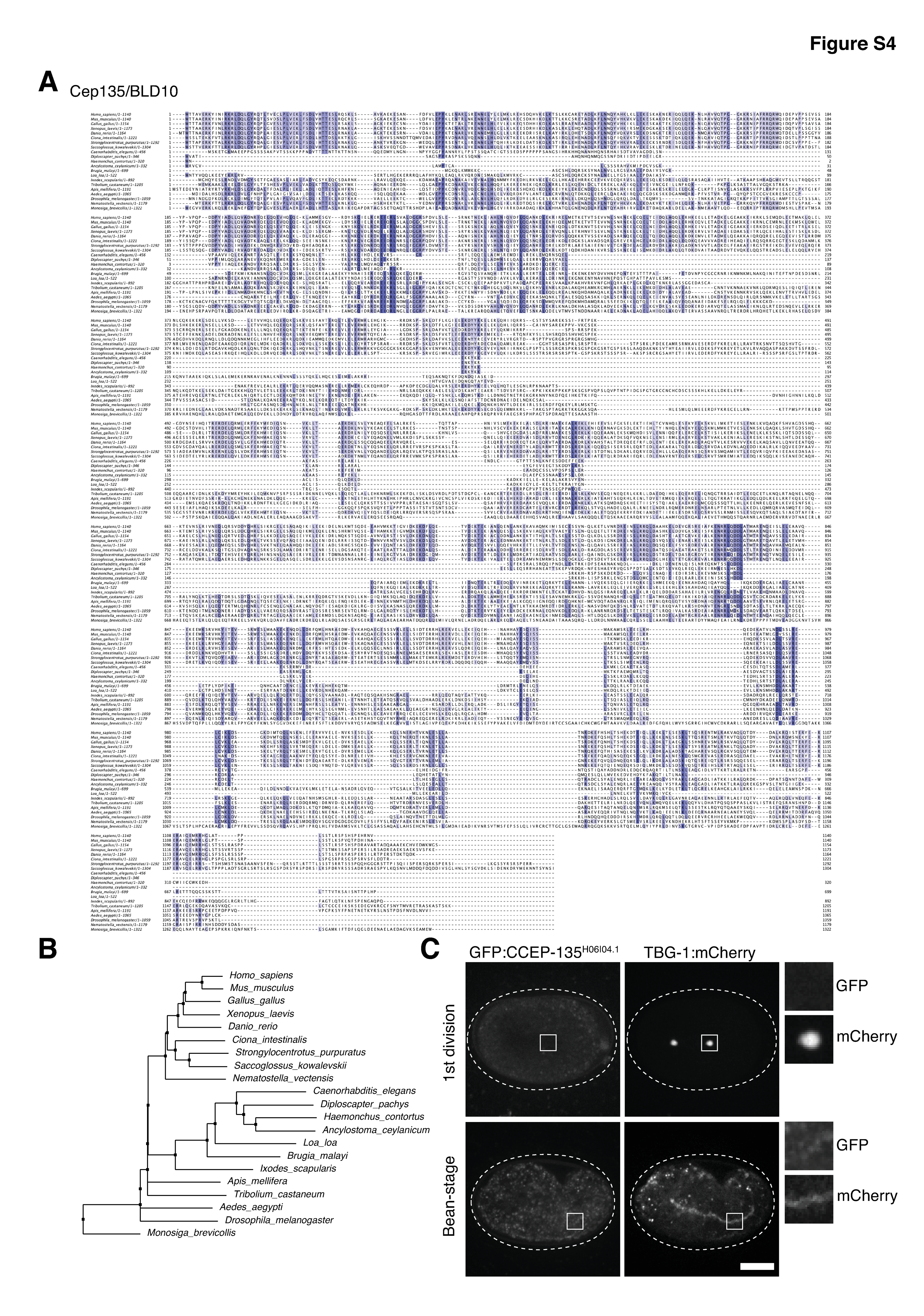

Supplement: S4 Fig — (A, B) Multiple sequence alignment (A) and neighbor joining phylogenetic tree (B) of selected Cep135 orthologs. Accession numbers are provided in S2 Table. Note that tree largely reflects pattern of evolutionary divergence. (C) Endogenous promoter GFP fusion of CCEP-135 does not localize to the mitotic centrosome in the early embryo or the non-centrosomal microtubule-organizing center in the intestinal primordium of bean-stage embryos (~360min after fertilization), both marked by TBG-1:mCherry. GFP images scaled to centrosomal signal at ciliary base in L1 stage larvae. Scale bar in C is 10μm. (TIF) [file pgen.1010150.s004.tif]
